# The microbial community in filamentous bulking sludge with the ultra-low sludge loading and long sludge retention time in oxidation ditch

**DOI:** 10.1038/s41598-019-50086-3

**Published:** 2019-09-23

**Authors:** Meng Zhang, Junqin Yao, Xiyuan Wang, Ying Hong, Yinguang Chen

**Affiliations:** 1 College of Resources and Environmental Science, Xinjiang University, Urumqi, 830046 China; 2 College of Environmental and Energy Engineering, Tongji University, Shanghai, 200092 China

**Keywords:** Water microbiology, Environmental impact

## Abstract

Sludge bulking is a major problem that restricts the development of the activated sludge process. The microbial community responsible for sludge bulking varies depending on water quality and operational conditions. This study analysed the microbial community of bulking sludge in oxidation ditch with ultra-low sludge loading and long sludge retention time using high-throughput sequencing. The study found that the relative abundance of bacterial genus *Saprospiraceae_norank* was the highest in bulking sludge, reaching 13.39–28.83%, followed by *Comamonadaceae_unclassified, Ardenticatenia_norank* and *Tetrasphaera*, with the relative abundance of 4.59–11.08%, 0.52–16.60% and 0.17–8.92% respectively. In contrast, the relative abundance of bacteria that easily caused sludge bulking including *Microthrix* (0.54–2.47%), *Trichococcus* (0.32–1.71%), *Gordonia* (0.14–1.28%), and *Thiothrix* (0.01–0.06%) were relatively low. *Saprospiraceae_norank* was predominant and induced sludge bulking in oxidation ditch. The relative abundance of fungal genus *Trichosporon* was the highest in bulking sludge, reaching 16.95–24.98%, while other fungal genera were *Saccharomycetales_unclassified* (5.59–14.55%), *Ascomycota_norank* (1.45–13.51%), *Galactomyces* (5.23–11.23%), and *Debaryomyces* (7.69–9.42%), whereas *Trichosporon* was the dominant fungal genus in bulking sludge. This study reported that excessive *Saprospiraceae_norank* can induce sludge bulking for the first time, which provides important knowledge to control sludge bulking.

## Introduction

Activated sludge process is widely applied in wastewater treatment plants (WWTPs) because of low investment, high treatment efficiency and strong adaptability^1^. It is reported that about 50% of WWTPs used oxidation ditch process in China^2^. Complete biodegradation in the aeration basin and good separation of mud and water in the secondary sedimentation tank are critical in the activated sludge process. However, sedimentation performance of activated sludge is often a problem. Sludge settling performance is generally measured using sludge volume index (SVI). Generally, good activated sludge has SVI of 50–150 mL/g, filamentous sludge bulking with poor sludge settling property occurs at an SVI over 150 mL/g, while severe sludge bulking occurs at SVI of greater than 250 mL/g^3^. There are two kinds of sludge bulking^4^: (1) filamentous sludge bulking induced by the mass proliferation of filamentous bacteria and (2) viscous sludge bulking caused by highly viscous substances produced by bacterial micelles^5^. Past research studies have reported that filamentous sludge bulking accounts for more than 90% of sludge bulking occurred in WWTPs and significantly affects the effluent quality and the operation and management of WWTPs^6^. Among several methods available to investigate sludge bulking, Gaussian Process Regression (GPR) is preferred since it can accurately predict and diagnose sludge bulking by monitoring the SVI values^7,8^.

Past studies have found that the main microbial communities responsible for sludge bulking in WWTPs are *Microthrix*^9^, *Eikelboom type 021N*^10^, *Flavobacterium*^11^, *Haliscomenobacter hydrossis*^12^, *Nocardia*^13^*, Thiothrix*^14^, *Tetrasphaera*^15^, *Trichococcus Nostocoida limicola I*^16^, *Beggiatoa*^17^, *Trichosporon*^18^, *Geotrichum*^19^, and *Penicillium*^20^ etc. Among them, the dominant bacterial communities that often induce sludge bulking in oxidation ditch are *Microthrix*^9^, *Flavobacterium*^21^*, Haliscomenobacter hydrossis*^12^, *Tetrasphaera*^15^, and *Trichococcus Nostocoida limicola I*^16^. Similarly, the dominant fungal communities that induce sludge bulking in oxidation ditch are *Trichosporon*^18^ and *Geotrichum*^19^.

Factors such as water temperature^22^, dissolved oxygen (DO)^3^, sludge retention time (SRT)^23^, pH^24^, influent quality^25^, nutrient ratio^26^ and sludge loading^27^ are responsible for filamentous sludge bulking. The microbial community responsible for sludge bulking varies depending on the water quality and operational conditions. For example, for bacterial communities, *Microthrix* proliferated at low sludge loading and low temperature^6,28^, whereas *Eikelboom type 021N* induced sludge bulking at high sludge loading and high temperature^10^. *Flavobacterium* proliferated and caused sludge bulking at low influent carbon/nitrogen(C/N) ratio and long hydraulic retention time (HRT)^11^. The mass propagation of *Haliscomenobacter hydrossis* caused sludge bulking and resulted in high sludge loading and long SRT^29^, whereas *Nocardia* induced sludge bulking when the sludge loading was less than 0.5 kg BOD_5_/(kg MLSS·d)^13^. *Thiothrix* proliferated and caused sludge bulking at high chemical oxygen demand(COD) concentration, low DO and low nutrient^30^. Excessive proliferation of *Tetrasphaera* and *Trichococcus Nostocoida limicola I* caused sludge bulking at low temperature^31^. Further, *Beggiatoa* proliferated and resulted in sludge bulking when the sludge loading was less than 0.51 kg BOD_5_/(kg MLSS·d) and the DO lower than 1.5 mg/L^17^. For fungal communities, excessive propagation of *Trichosporon* caused sludge bulking at low DO^18^, while *Geotrichum* caused sludge bulking at low pH and high temperature^19^.

Although filamentous bacteria causing sludge bulking under different operational conditions have been widely investigated, sludge bulking is still a major problem hindering the operation of the activated sludge process. High-throughput sequencing is a revolutionary reform to traditional sequencing since the former does not require a pure culture and can sequence hundreds of thousands to millions of deoxyribonucleic acid (DNA) molecules rapidly and accurately^32^. In this study, the microbial community in the sludge collected from an oxidation ditch that has been experiencing sludge bulking constantly in recent years was analysed using high-throughput sequencing technology with the ultra-low sludge loading and long SRT. The outcomes of this study are expected to provide valuable knowledge required to control the sludge bulking.

## Results

### Treatment efficiency of the WWTP

The removal efficiencies of the WWTP from January 2016 to January 2018 are presented in Table 1. The average influent biochemical oxygen demand (BOD) to COD ratio was 0.46, indicating a good biochemical property of sewage. The COD, BOD_5_, suspended solids (SS), total phosphorus (TP), and total nitrogen (TN) in the design influent to the WWTP were 450 mg/L, 200 mg/L, 250 mg/L, 5 mg/L, and 40 mg/L. As evident in Table 1, the actual influent COD, BOD_5_, SS, TP, and TN concentrations during the sampling period were 2–3 times higher than the design influent concentrations in the WWTP. The transformation of influent TN may generate substantial levels of free ammonia and free nitrous acid, which can have adverse impacts on microbial community^33,34^. The effluent from the WWTP met the second-level discharge standard^35^. Despite the sludge bulking, the sewage treatment efficiency was good.

**Table 1:** Treatment effciencies of the WWTP.

Indicator	Influent (mg/L)	Effluent (mg/L)	Removal efficiency (%)
COD	715~1233	66~92	91~94
BOD_5_	265~625	17~27	91~96
SS	267~490	9~27	92~98
TN	66~94	4~31	58~93
TP	5.4~14.2	0.1~1.9	81~99

### Activated sludge settling property

**Figure 1:**
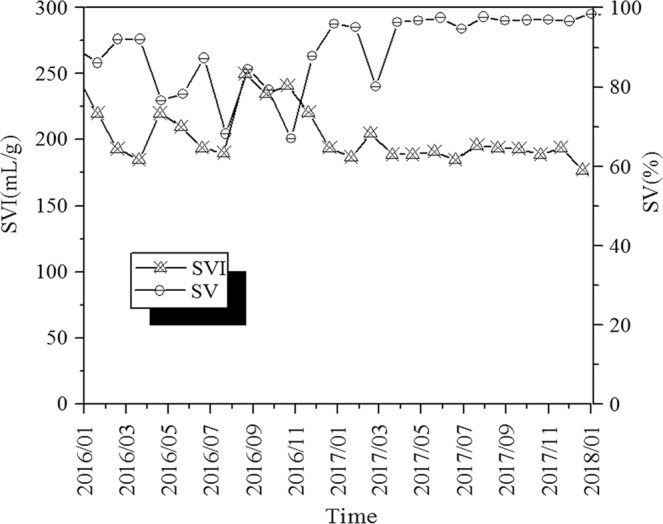
Variation tendency of the SVI and SV% value of the WWTP.

As shown in Fig. 1, SVI of the activated sludge samples were 162–250 mL/g indicating poor settling property and the activated sludge in the oxidation ditch was in the state of constant sludge bulking. Furthermore, filamentous sludge bulking occurred in the WWTP according to the microscopic examination.

### Bacterial community analysis based on 16S rRNA sequencing

The total effective readings of the seven bulking sludge samples were between 30457 and 55170. The coverage indexes of all samples were more than 0.988, indicating the detection of most bacterial communities in this sequencing with high data reliability. The operational taxonomic units (OTUs), Chao, Shannon values are presented in Table 2. The Chao and Shannon indexes represent the richness and diversity of the microbial community, respectively. Higher Chao index indicates higher species richness and higher Shannon index suggests higher diversity of the communities^36^. In January 2016 (CJ1), the SVI value was the largest, while the Chao and Shannon values were the lowest, suggesting the lowest richness and diversity of the bacterial community. In January 2018 (CJ7), SVI value was the lowest, whereas the Chao and Shannon values were the highest, indicating the highest richness and diversity of the bacterial community. The richness and diversity of the bacterial community are lower when significant sludge bulking occurred.

**Table 2:** Values of OTUs, Chao, Shannon of bacterial community.

Sample	Reads	OTU	Chao	Shannon	Coverage
CJ1	46680	851	1041	4.914	0.990
CJ2	55170	866	1059	5.159	0.990
CJ3	30457	931	1084	5.107	0.988
CJ4	42278	979	1230	5.242	0.988
CJ5	41208	954	1183	5.185	0.989
CJ6	40215	978	1201	5.192	0.989
CJ7	48366	1031	1234	5.267	0.988

**Figure 2:**
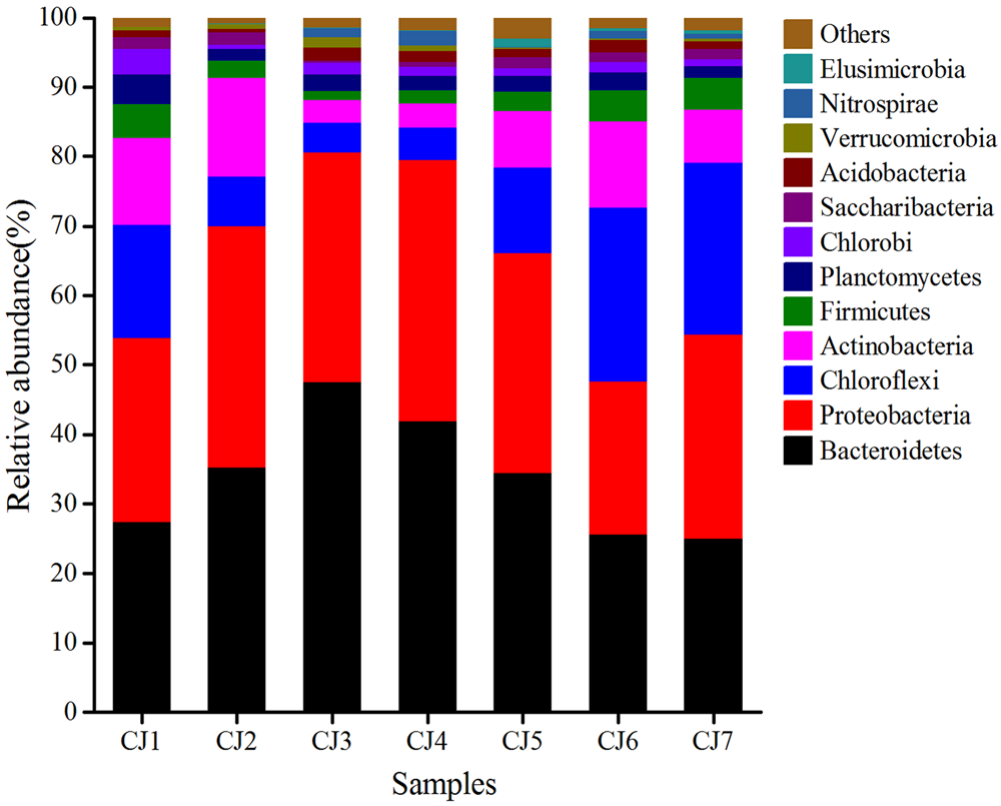
Variation in the relative abundance of bacterial phylum in bulking sludge samples.

A total of 35 bacterial phyla were detected in seven sludge samples. In at least one sample, there were 12 bacterial phyla with relative abundances of over 1%, accounting for 97.05–99.25% of the total bacterial effective sequences (Fig. 2). The dominant bacterial phyla were Bacteroidetes (25.86–47.56%), Proteobacteria (21.98–37.77%), Chloroflexi (4.28–24.96%), Actinobacteria (3.29–14.12%), and Firmicutes (1.20–4.65%).

**Figure 3:**
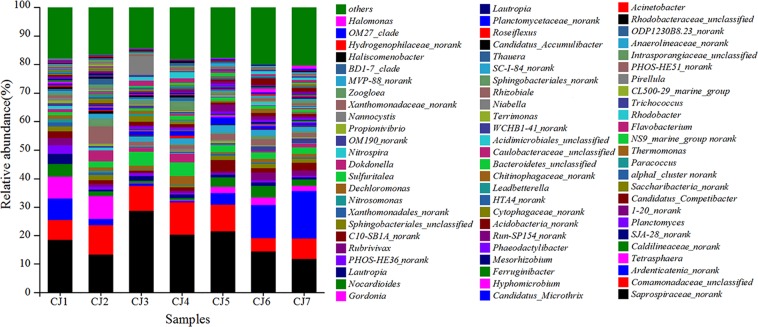
Variation in the relative abundance of bacterial genus in bulking sludge samples.

531 bacterial genera were present in seven sludge samples. In at least one sample, there were 73 bacterial genera with relative abundances of over 0.5%, accounting for 78.32–83.21% of the total bacterial effective sequences (Fig. 3). The dominant bacterial genera observed included *Saprospiraceae_norank* (11.87–28.83%), *Comamonadaceae*_*unclassified* (4.59–11.08%), *Ardenticatenia_norank* (0.52–16.60%) and *Tetrasphaera* (0.17–8.92%). The relative abundance of filamentous bacteria related to sludge bulking such as *Microthrix* (0.54–2.47%), *Trichococcus* (0.32–1.71%), *Gordonia* (0.14–1.28%) and *Thiothrix* (0.01–0.06%) was relatively low, among which *Saprospiraceae_norank* was predominant in all bacterial genera.

### Fungal community analysis based on 18S rRNA sequencing

The total effective readings of the three bulking sludge samples were between 31191 and 50116. The coverage indexes of all samples achieved 1.000, suggesting that the fungal community was detected in this sequencing. The OTUs, Chao, Shannon values are summarized in Table 3. In December 2016 (CJ3), SVI value was the largest, while the Chao and Shannon values were the lowest, suggesting the lowest richness and diversity of the fungal community. In January 2018 (CJ7), the SVI value was the smallest, whereas the Chao and Shannon values were the highest, indicating that the highest richness and diversity of the fungal community. The richness and diversity of the fungal community are generally lower for significant sludge bulking to occur.

**Table 3:** Values of OTUs, Chao, Shannon of fungal community

Samples	Reads	OTU	Chao	Shannon	Coverage
CJ3	41229	80	84	2.908	1.000
CJ6	31191	103	106	2.946	1.000
CJ7	47903	115	119	3.032	1.000

**Figure 4:**
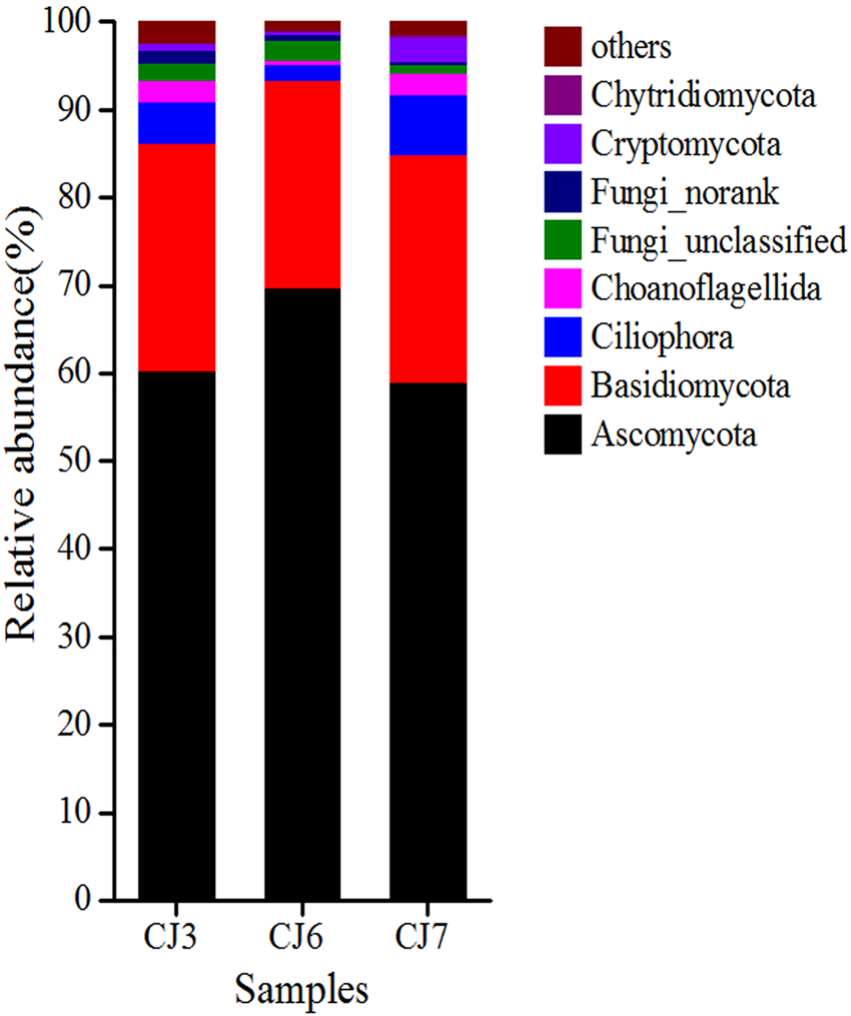
Variation of relative abundance of fungal phylum in bulking sludge samples.

A total of 22 fungal phyla were detected in three sludge samples. In at least one sample, there were 8 fungal phyla with relative abundances of over 1%, accounting for 97.56–99.01% of the total fungal effective sequences (Fig. 4). The dominant fungal phyla were Ascomycota (58.83–69.69%) and Basidiomycota (23.71–25.68%), while other phyla included Ciliophora (1.74–7.02%), Choanoflagellida (0.55–2.52%), Cryptomycota (0.39–2.77%), and Chytridiomycota (0.06–0.43%).

**Figure 5:**
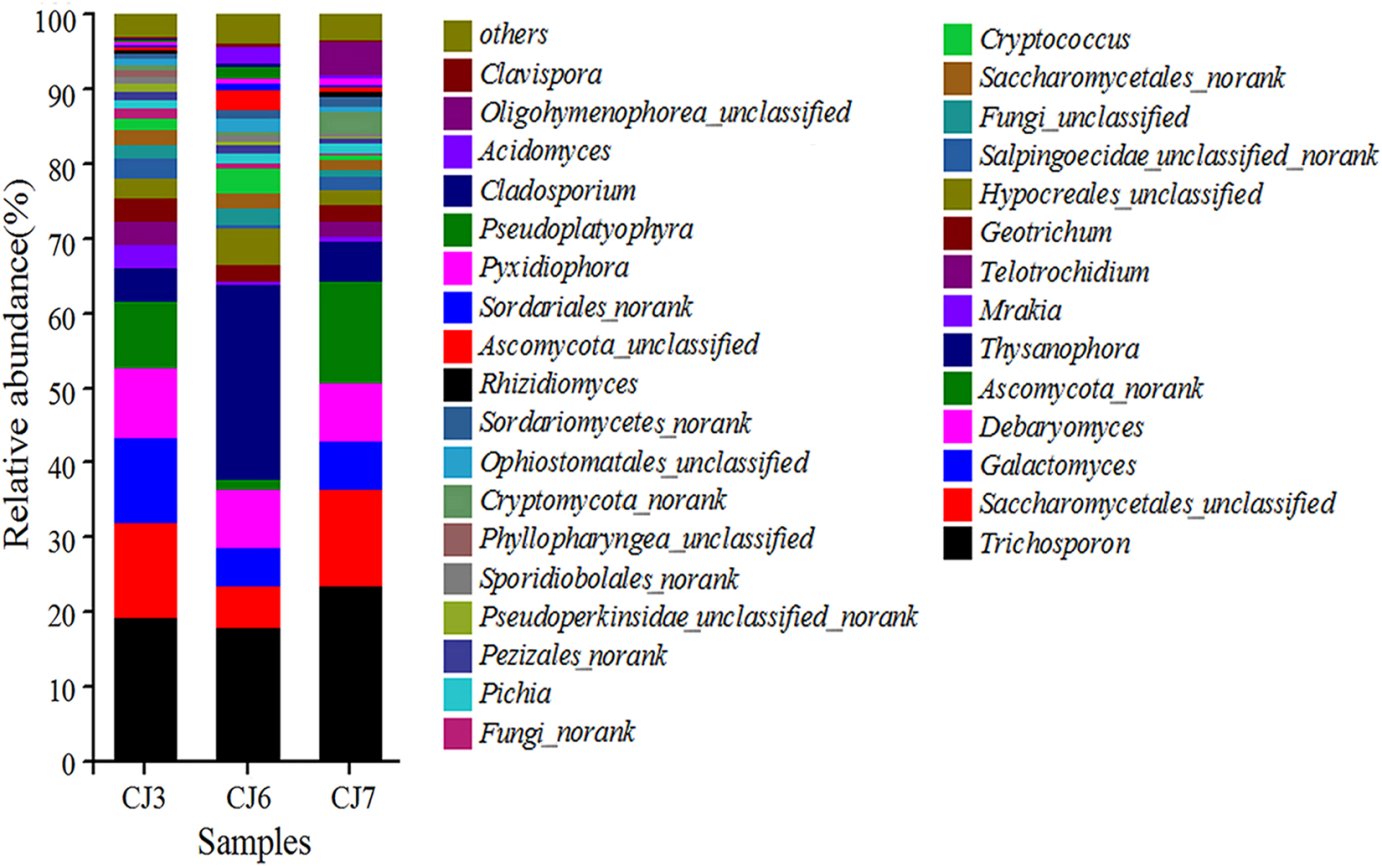
Variation of relative abundance of fungal genus in bulking sludge samples.

89 fungal genera were present in three sludge samples. In at least one sample, there were 32 fungal genera with relative abundances of over 0.5%, accounting for 96.34–97.12% of the total fungal effective sequences. Fungi are less common than bacteria, though more evenly distributed than bacteria (Fig. 5). The top five dominant fungi in the relative abundance of bulking sludge samples were *Trichosporon* (16.95–24.98%), *Saccharomycetales_unclassified* (5.59–14.55%), *Ascomycota_norank* (1.45–13.51%), *Galactomyces* (5.23–11.23%), and *Debaryomyces* (7.69–9.42%), among which *Trichosporon* was predominant in all fungal genera.

## Discussion

The dominant bacterial phyla obtained were Bacteroidetes (25.86–47.56%), Proteobacteria (21.98–37.77%), Chloroflexi (4.28–24.96%), and Actinobacteria (3.29–14.12%) and are not significantly different from the dominant bacterial phyla in the oxidation ditch process of WWTP in China^37–39^. However, the relative abundance of these phyla is different. Bacteroidetes, which plays an important role in wastewater treatment by degrading macromolecular organic pollutants^40^, were present at 25.86–47.56% in bulking sludge samples, and were the dominant bacterial phylum. Kragelund *et al*. found that the relatively high abundance of Bacteroidetes can cause sludge bulking problems^41^. Proteobacteria, which is a conventional bacterial phylum in WWTPs with the ability to degrade organic pollutants and remove nutrients such as biological nitrogen and phosphorus^42^, were present at 21.98–37.77% in all bulking sludge samples. Xu *et al*. found that Proteobacteria (33.90–50.90%) was the dominant bacterial phylum in the oxidation ditch without sludge bulking^38^. Chloroflexi is chiefly filamentous bacteria, which exists in flocculent sludge clump inside the body in the form of flocs skeleton. It plays a role in sludge flocculation, but rarely induces sludge bulking^43^. The relative abundance of Chloroflexi was between 4.28% and 24.96% in all bulking sludge samples. Furthermore, the mass proliferation of Actinobacteria can cause filamentous sludge bulking^6^. Wang *et al*. found that Actinobacteria was dominant with a relative abundance of 50% in the WWTP, where excessive sludge bulking occurred^31^. In this study, the relative abundance of Actinobacteria (3.29–14.12%) was low and it was not the main bacterial phylum in bulking sludge of the WWTP.

The dominant bacterial genera obtained were *Saprospiraceae_norank* (11.87–28.83%), *Comamonadaceae_unclassified* (4.59–11.08%), *Ardenticatenia_norank* (0.52–16.60%), and *Tetrasphaera* (0.17–8.92%). Martins *et al*. reported that *Microthrix* was the dominant filamentous bacterial genus that caused sludge bulking, despite the different operational conditions in different WWTPs^25^. *Microthrix* (15.11%) was the dominant filamentous bactarial genus at low temperature in WWTP in China^31^. Miłobędzka *et al*. studied the filamentous bacteria of WWTPs in Poland and found that *Microthrix* (25%) was dominant with the long SRT^12^. Madoni *et al*. reported that excessive growth of *Microthrix* (53.20%) resulted in sludge bulking when the sludge loading was 0.1–0.2 kgBOD_5_/(kg MLVSS·d)^44^. Knoop *et al*. reported that *Microthrix* has a strong reproductive advantage at low temperature (≤12–15 °C) and is the dominant bacterial genus  responsible for sludge bulking in cold areas^28^. Xinjiang is a dry and cold region with the winter lasting for five months. The influent temperature remains at 7–15 °C in winter and 16–24 °C in summer in the WWTP. However, in this study, *Saprospiraceae_norank* was the predominant bacterial genus in oxidation ditch bulking sludge and its relative abundance varied between 11.87% and 28.83%, whereas Yang *et al*. found that the relative abundance of *Saprospiraceae_norank* was between 2.10% and 3.53% in non-bulking activated sludge in WWTP^21^. Muszynski *et al*. reported that the abundance of *Saprospiraceae_norank* was dependent on the season^45^. Additionally, *Saprospiraceae_norank*, which existed in sludge flocs and is capable to produce extracellular enzymes to degrade protein and is crucial for partial nitrification, denitrification and sludge fermentation^46^. *Saprospiraceae_norank* belongs to phylum Bacteroides, class *Sphingoleifera*, order *Sphingoleiferae*, and family *Saprospiraceae*. Shchegolkova *et al*. found that *Saprospiraceae_norank* was the inductor of activated sludge bulking and foaming^47^. Yao *et al*. found that sludge bulking was inhibited due to the addition of an anaerobic step^48^. In this study, *Saprospiraceae_norank* was the predominant bacterial genus that induced sludge bulking in oxidation ditch, while the relative abundance of *Microthrix* was only between 0.54% and 2.47% in bulking sludge, and was far less than the relative abundance reported when the proliferation of *Microthrix* caused sludge bulking in WWTPs. *Microthrix* was not the dominant bacterial genus that caused sludge bulking in oxidation ditch in WWTP.

*Tetrasphaera* belongs to Actinobacteria and plays a role in the biological phosphorus removal in WWTPs^49^. According to Wang *et al*., *Tetrasphaera* (6.75%) was generally found in activated sludge systems, where filamentous sludge bulking occurred at low temperature and contributed to sludge bulking in WWTPs^31^. The relative abundance of *Tetrasphaera* was between 0.17% and 8.92% in all bulking sludge samples. *Trichococcus* (3.91%) was the dominant filamentous bacterial genus that caused sludge bulking in WWTP at low temperature and low DO^16,31^. The relative abundance of *Trichococcus* (0.32–1.71%) was low and was not the dominant filamentous bacterial genus in oxidation ditch bulking sludge. Similarly, *Gordonia* (5.1%) was the dominant bacterial genus in WWTPs with low DO, long SRT and low temperature^16^. A long-term study that was conducted to identify the dominant filamentous bacteria in a full-scale WWTP found that *Thiothrix* (51.9%) was the dominant filamentous bacterial genus with the high COD concentration, low DO and nutrient deficits^30^. However, in this study, the relative abundance of *Gordonia* (0.14–1.28%) and *Thiothrix* (0.01–0.06%) were low and were not the dominant filamentous bacterial genera in oxidation ditch bulking sludge. Speirs *et al*. studied the bacterial community structure in oxidation ditch of a WWTP with severe sludge bulking in South Australia and found that the dominant bacterial genus was *Type 0914* (35%) with the long SRT^50^. Dos Santos *et al*. studied the dominant filamentous bacteria in WWTPs in Portugal and found that *Type 0041/0675* (19%) and *Type 0092* (14%) induced sludge bulking with the low sludge loading and long SRT^29^, while *Type 0914, Type 0041/0675* and *Type 0092* were not detected in all bulking sludge samples. The dominant filamentous bacteria in WWTPs with sludge bulking in different regions, water quality and operational conditions are listed in Table 4. Though Past studies did not report that excessive proliferation of *Saprospiraceae_norank* can induce sludge bulking, this study found that excessive *Saprospiraceae_norank* can induce sludge bulking for the first time.

**Table 4:** Filamentous bacteria of WWTPs with sludge bulking in different regions, water quality and operational conditions.

Geographical names	Dominant filamentous bacteria	Main reasons	Reference
China	*Microthrix*, *Tetraspharea*, *Trichococcus*	Low DO, low temperature and high influent NH_4_^+^-N concentration	^15, 31^
Australia Poland	*Type 0914*, *Microthrix*	Long SRT	^12, 50^
Portugal (16 activated sludge systems), Italy	*Type0041/0675/0092*, *Microthrix*	Long SRT and low sludge loading	^29, 44^
Germany	*Microthrix*, *Gordonia*, *Thiothrix*	Low DO, long SRT, low temperature, high COD concentration	^16, 30^

Ascomycota (58.83–69.69%) and Basidiomycota (23.71–25.68%) were the dominant fungal phyla obtained by fungal sequencing in WWTP, which is consistent with the results obtained in other WWTPs in China. Ascomycota and Basidiomycota were conventional fungal phyla in activated sludge of WWTPs and Ascomycota (51.82%) was dominant in all fungal phyla^51^. Basidiomycota mainly affects the formation of sludge flocs by reducing sludge settling property^52^ and thereby inducing sludge bulking. In this study, Basidiomycota was the dominant fungal phylum that caused sludge bulking in the oxidation ditch.

*Trichosporon* (16.95–24.98%) was the dominant fungal genus obtained by fungal sequencing and was responsible for inducing sludge bulking^53^, whereas *Trichosporon* (25%) was the dominant fungal genus when sludge bulking occurred in WWTP^18^. This is consistent with the dominant fungal genus obtained in this study. Further, *Trichosporon* induced sludge bulking in a sequencing batch reactor (SBR) in a previous laboratory study^14^. The study found that *Trichosporon* was the filamentous fungal genus causing sludge bulking under different operational conditions. *Saccharomycetales_unclassified* (5.59–14.55%) and *Debaryomyces* (7.69–9.42%) are common yeasts found in the oxidation ditch of WWTPs in China^54^. *Saccharomycetales_unclassified* and *Debaryomyces* can produce hydrolytic enzymes and collectively degrade some pollutants in sewage^55^. In this study, *Saccharomycetales_unclassified* and *Debaryomyces* existed in all bulking sludge in the oxidation ditch of the WWTP. However, they are not filamentous fungi and do not generally induce sludge bulking. *Galactomyces* is a common filamentous fungal genus in activated sludge of WWTPs and can induce sludge bulking^20^. Comparatively high relative abundance of *Galactomyces* (5.23–11.23%) in oxidation ditch bulking sludge could contribute to the sludge bulking in WWTP, while the relative abundance of *Geotrichum*^19^ related to sludge bulking was low, only 2.17–3.02%. In other words, *Trichosp*oron was the dominant filamentous fungal genus when sludge bulking occurred in WWTP, which was consistent with past study results. In this study, sludge bulking in WWTP occurred due to high influent pollutant concentrations, ultra-low sludge loading and long SRT. They are discussed in detail below.

1. **High influent pollutant concentrations**. The influent quality (COD, BOD_5_, SS, TN, and TP) of the WWTP was generally higher than that of other WWTPs in China, where the influent pollutant concentration is generally high. China has 656 domestic sewage treatment plants in 70 cities including Shenyang, Changchun, Dalian, Harbin, Tianjin, Shanghai, Beijing, Wuhan, Zhengzhou, Shijiazhuang, etc^2^. The average influent COD, BOD_5_, SS, TN, and TP concentrations in these WWTPs were 395 mg/L, 220 mg/L, 250 mg/L, 55 mg/L, and 8.7 mg/L, respectively. In this study, the average influent COD, BOD_5_, SS, TN, and TP concentrations in WWTP from January 2016 to January 2018 were 938 mg/L, 441 mg/L, 377 mg/L, 77 mg/L, and 10.6 mg/L, respectively. High influent pollutant concentration may be one of the reasons for sludge bulking in oxidation ditch.
2. **Ultra-low sludge loading operation**. From January 2016 to January 2018, the SVI values and sludge loading change trend of WWTP are shown in Fig. 6. In January and September 2016, the sludge loading were the lowest, only 0.007 kg COD/(kg MLSS·d), while the SVI values were the highest, reaching 250 mL/g. In January 2018, the maximum sludge loading was 0.016 kg COD/(kg MLSS·d), with the minimum SVI value of 177 mL/g. The SVI value decreased with the increase in sludge loading. High, medium and low sludge loadings were 0.12 ± 0.016 kg COD/(kg MLSS·d), 0.07 ± 0.015 kg COD/(kg MLSS·d), and 0.04 ± 0.004 kg COD/(kg MLSS·d) respectively^56^. However, in this study, the sludge loading was less than 0.02 kg COD/(kg MLSS·d) due to ultra-low sludge loading operation. Sludge bulking occurs when the activated sludge system operates under low sludge loading condition for a long time.

**Figure 6:**
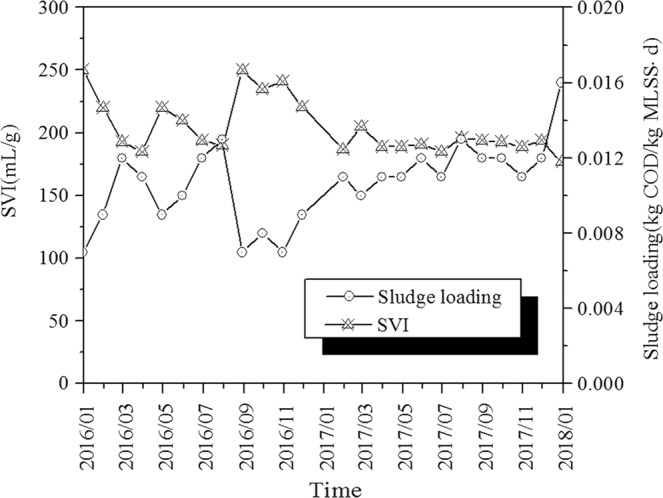
Variation tendency of the SVI value and Sludge loading of the WWTP.
3. **Long SRT**. The design SRT of oxidation ditch in WWTP was 25 d, while the actual sludge retention time was 40 d. The particle size distribution of sludge floc and sludge settling property are directly affected by increasing SRT. Settling test conducted on activated sludge with sludge retention time ranging from 0.25 to 12 days showed that increased SRTs resulted in an exponential decrease in percent dispersion (non-flocculent or pinpoint floc), and an increase in the particle size of the floc, leading to a good sludge settling property. However, SRTs greater than 12 d resulted in a reduce in the diameter of the floc and the decline in sludge settling property^57^. Significantly long SRT can lead to poor sedimentation performance of activated sludge, resulting in further sludge bulking.

## Materials and Methods

### Description of the WWTP and sample collection

The WWTP located in the Changji city of Xinjiang, northern China, with the treatment design capacity of 10 × 10^4^ m^3^/d uses the carrousel oxidation ditch process and has been operating since 2000. The influent of Changji WWTP is mainly domestic wastewater. Activated sludge samples, CJ1, CJ2, CJ3, CJ4, CJ5, CJ6, and CJ7, were collected from the end of the aerobic stage of the oxidation ditch. Sampling date, sludge index volume and operating parameters of WWTP are presented in Table 5. The SVI values of the samples were more than 150 mL/g, confirming that all samples were bulking sludge.

**Table 5:** Sampling date, sludge settling property, sludge concentration and water temperature of the operation.

Sample	Sampling date	MLSS (mg/L)	SVI (mL/g)	SV(%)	Water temperature (°C)
CJ1	2016.01.25	3355	262	88	13.7
CJ2	2016.03.02	4091	220	90	13.5
CJ3	2016.12.23	4265	211	90	15.2
CJ4	2017.03.31	5107	192	98	13.5
CJ5	2017.07.26	5132	187	96	24.5
CJ6	2017.11.24	5092	189	96	16.3
CJ7	2018.01.23	5279	188	99	11.7

### Analysis methods

COD, BOD_5_, TN, TP, SS and mixed liquor suspended solids (MLSS) were assayed according to the standard method^58^. The temperature was measured using a thermometer. SVI values were determined by reading the percentage of sludge volume in the mixture of water and sludge after 30 min settling in a 100 mL measuring cylinder and counted from the dry weight in MLSS. Microscopic examination was conducted using a photonic microscope. The morphology of activated sludge filaments and flocs was characterized daily.

### DNA extraction and PCR amplification

The E.Z.N.A. soil DNA Kit (Omega Bio-Tek, Norcross, GA, USA) was used for microbial DNA extraction from eight samples according to the manufacturer’s instructions. The NanoDrop 2000 UV-vis spectrophotometer (Thermo Scientific, Wilmington, USA) was used for measuring the final DNA concentration and purification, while the quality of the DNA was checked by 1% agarose gel electrophoresis. The V4-V5 hypervariable regions of 16S rRNA gene of all samples were amplified with primers 515F(5′-GTGCCAGCMGCCGCGG-3′) and 907R(5′-CCGTCAATTCMTTTRAGTT T-3′), while the fungal 18S rRNA gene of three sludge samples (CJ3, CJ6 and CJ7) were amplified with primers SSU 0817F(5′-TTAGCATGGAATAATRRAATAGGA-3′) and 1196R(5′-TCTGGACCTGGTGA GTTTCC-3′) by thermocycler PCR system (GeneAmp 9700, ABI, USA). The PCR reactions were executed using 20 μL reaction mixtures, containing 4 μL of 5 × FastPfu Buffer, 2 μL of 2.5 mM dNTPs, 0.8 μL of each primer (5 μM), 0.4 μL of FastPfu Polymerase and 10 ng of template DNA. The triplicate amplicons were pooled together for each sample. The AxyPrep DNA Gel Extraction Kit (Axygen Biosciences, Union City, CA, USA) was used together with the quantification using QuantiFluor-ST (Promega, USA) for the extraction of PCR products from a 2% agarose gel and for further purification according to the manufacturer’s introductions.

### High-throughput sequencing and Data analysis

Purified amplicons were pooled in equimolar and paired-end sequenced (2 × 300) on an Illumina MiSeq platform (Illumina, San Diego, USA) according to the standard introductions of Majorbio Bio-Pharm Technology Co. Ltd. (Shanghai, China). The raw readings were deposited into the NCBI Sequence Read Archive (SRA) database (Accession Number: SRP197666). The i-Sanger platform (http://www.i-sanger.com/) was provided by Majorbio Bio-Pharm Technology Co. Ltd (Shanghai, China) for conducting data analysis. The Chao estimator and the Shannon diversity index were used to calculate the microbial phylotype richness levels. The Mothur program version v.1.30.1 (http://www.mothur.org/wiki/Schloss_SOP#Alpha_diversity) was used to calculate the Chao estimator, the Shannon diversity index, and the coverage percentage. These analyses were performed using the R Programming Language software.
